# Effect of lactobacilli inoculation on protein and carbohydrate fractions, ensiling characteristics and bacterial community of alfalfa silage

**DOI:** 10.3389/fmicb.2022.1070175

**Published:** 2022-12-05

**Authors:** Wenjie Huo, Yujuan Zhang, Luyao Zhang, Chen Shen, Lei Chen, Qiang Liu, Shuanlin Zhang, Cong Wang, Gang Guo

**Affiliations:** Animal Sciences College, Shanxi Agricultural University, Taigu, Shanxi, China

**Keywords:** alfalfa silage, lactobacilli inoculants, ensiling characteristics, protein fractions, bacterial community

## Abstract

**Introduction:**

Alfalfa (*Medicago sativa* L.) silage is one of the major forages with high protein for ruminants.

**Methods:**

The objective of this study was to investigate the effects of lactobacilli inoculants on protein and carbohydrate fractions, ensiling characteristics and bacterial community of alfalfa silage. Wilted alfalfa (35% dry matter) was inoculated without (control) or with *Lactobacillus coryniformis*, *Lactobacillus casei*, *Lactobacillus plantarum*, and *Lactobacillus pentosus* and ensiled for 7, 15, and 60 days.

**Results and discussion:**

Silage inoculated with *L. pentosus* was superior to *L. coryniformis*, *L. casei*, *L. plantarum* in improving the fermentation quality of alfalfa silage, as indicated by the lowest ammonia nitrogen content and silage pH during ensiling. There was minor difference in water soluble carbohydrates content among all silages, but *L. pentosus* inoculants was more efficient at using xylose to produce lactic acid, with lower xylose content and higher lactic acid content than the other inoculants. Compared with the control, *L. pentosus* inoculants did not affect true protein content of silage, but increased the proportions of buffer soluble protein and acid detergent soluble protein. The *L. pentosus* inoculants reduced the bacterial diversity In alfalfa silage with lower Shannon, Chao1, and Ace indices, and promoted relative abundance of *lactobacillus* and decreased the relative abundance of *Pediococcus* compared with the control. As well as *L. pentosus* inoculants up-regulated amino acid, carbohydrate, energy, terpenoids, and polypeptides metabolism, and promoted lactic acid fermentation process. In summary, the fermentation quality and nutrient preservation of alfalfa silage were efficiently improved by inoculated with *L. pentosus.*

## Introduction

With the rapid development of ruminant husbandry, alfalfa (*Medicago sativa* L.) becomes an essential roughage for dairy ration. Ensiling is one of the effective methods to preserve alfalfa nutrients (Ertekin and Kizilsimsek, 2020). During ensiling, most true protein (TP) fractions in alfalfa are degraded into non-protein nitrogen (NPN). In particular, the activity of harmful microorganisms, such as clostridia and enterobacteria, extensively reduce the amino acids content, leading to nutrient losses (Buxton et al., 2003). In the past few decades, numerous researchers have reduced proteolysis of alfalfa silage by inoculating with lactobacilli, which can rapidly reduce silage pH and future prevent activities of plant proteolytic enzymes and harmful microorganisms, and effectively reduce NPN, especially ammonia formation (Oliveira et al., 2017; Tian et al., 2017; Kung et al., 2018). Homofermentative lactobacilli rapidly reduce alfalfa silage pH is more effective than heterofermentative lactobacilli (Contreras-Govea et al., 2011), and *Lactobacillus plantarum* is commonly used. In fact, *L. plantarum* is a facultative heterofermentative lactic acid bacteria (LAB), which produce acetic acid and reduce the acidification efficiency of silage under the insufficient hexose, and alfalfa is usually low in hexose (Buxton et al., 2003). However, little attention has been paid to comparing the effects of different homofermentative lactobacilli species on nitrogen distribution of alfalfa silage.

The efficacy of LAB inoculants is influenced not only by its phenotypic characteristics, but also by chemical and microorganism characteristics of pre-ensiling material (Tohno et al., 2012). Similarly, changes in chemical composition of silage are also inextricably linked to changes in microbial communities during ensiling. Therefore, it is essential for thoroughly understand and improve the fermentation quality of silage to monitor the relationship between changes in chemical composition and microbial communities during ensiling process (Namihira et al., 2010; Wang et al., 2020). In addition, it is difficult to evaluate the nitrogen distribution of alfalfa silage based on the traditional feed nutrition assessment system, while the Cornell’s net carbohydrate and protein system could reflect the nutritional components of feed more comprehensively and objectively (Wang et al., 2009).

Thus, the purpose of current study was to investigate the effects of homofermentative lactobacilli (*Lactobacillus coryniformis*, *Lactobacillus casei*, and *Lactobacillus pentosus*) and facultative heterofermentative lactobacilli (*L. plantarum*) on the fermentation quality and nutrient preservation of alfalfa silage, based on ensiling characteristics, protein fractions, carbohydrate fractions and bacterial diversity and functional analysis.

## Materials and methods

### Silage preparation

Alfalfa (*Medicago sativa* L., cultivar SR 4030) planted for 2 years was harvested at budding stage on May 15, 2021, and wilted to about 35% dry matter (DM) in the experimental field (37°25′08″N, 112°35′25″E, elevation 783 m) of Shanxi Agricultural University (Taigu, China). The wilted alfalfa was chopped into approximately 20 mm and divided into 60 sub-samples (2.5 kg per sub-sample), which were assigned to the following ensiling treatments in four replicates: (1) Control (sprayed with equal distilled water, C); (2) *L. coryniformis* (LCO); (3) *L. casei* (LCA); (4) *L. plantarum* (LPL) and (5) *L. pentosus* (LPE). Each treatment was sprayed onto the forage with a mini-sprayer at a rate of 1.0 × 10^6^ cfu/g fresh weight (FW). After thoroughly mixing, 500 g of the treated forage was packed into a laboratory sterile plastic bag with a release valve, vacuum-sealed and stored at room temperature for 7, 15, and 60 days.

### Chemical composition and microbial amount analyses

Each silage sample was weighed (20 g) and diluted with twice the volume of distilled water for 24 h at 4°C, and then filtered through two layers of cheesecloth and a Whatman filter paper (Desta et al., 2016). The filtrate was used for determining pH, ammonia nitrogen (ammonia-N), lactic acid and volatile fatty acid (VFA, acetic, propionic and butyric acids) concentrations. Silage pH was measured by pH meter (pH FE28, Mettler-Toledo Instruments Co., Ltd., China). Ammonia-N and lactic acid were determined according to the method of Kleinschmit et al. (2005), and VFA concentrations were determined by gas chromatography with 30 m × 0.25 mm (df 0.25 μm) capillary column (Thermo T1300, United States; Guo et al., 2019).

DM content of raw material and silage were determined at 65°C for 48 h in an oven with forced-air circulation. Total nitrogen (TN) and ash contents were determined according to AOAC (2012). Organic matter (OM) content was estimated as DM minus ash, and CP content was calculated by multiplying TN by 6.25. Neutral detergent fiber (NDF), Acid detergent fiber (ADF), acid detergent lignin (ADL), neutral detergent insoluble protein (NDIP) and acid detergent insoluble protein (ADIP) contents were determined with the method of VanSoest et al., 1991. Water soluble carbohydrates (WSC) content was determined with the method of Kim and Adesogan (2006). Starch content was determined with an enzymatic method (Owens et al., 2002). Xylose content was determined with the method of Eberts et al. (1979). The TP and NPN contents were determined with the method of Licitra et al. (1996). The Cornell net carbohydrate and protein system was used to estimate the protein fractions of raw material and silage (Nadeau et al., 2012). Buffering capacity of raw material was analyzed by the method of Playne and McDonald (1966). Water activity was determined by water activity analyzer (HD-3A, Ester Instrument Co., Ltd., Xiamen, China).

Each sample (10 g) was mixed with 90 ml of sterilized saline solution (0.85% NaCl), and serially diluted with 10^−1^ and 10^−8^. The dilutions were used to analyze microbial counts with the pour plate method. The LAB were counted using the de Man, Rogosa and Sharpe agar plates (Basebio, Co., Ltd., Hangzhou, China) in an anaerobic box (TE-HER Hard Anaerobox, Hirosawa Ltd., Tokyo, Japan) at 35°C for 48 h. Total aerobic bacteria were counted on nutrient agar plates (Basebio, Hangzhou, China), and yeasts and molds were counted on rose bengal agar plates (Basebio, Hangzhou, China). The agar plates were maintained in an incubator at 30°C for 48 h (Cai et al., 1999).

### Microbial community analysis

Each silage sample (20 g) was mixed with 180 ml of sterilized saline solution. Extracts were shaken at 4°C for 2 h (150 rpm/min), and filtered through two layers of cheesecloth. Then, pellets were obtained by centrifugation at 12000 rpm/min for 15 min at 4°C. Total DNA was extracted using TIANamp Bacteria DNA Kit (DP302-02, Tiangen, Beijing, China). The PCR was used to amplify the V3-V4 region of the bacterial 16S rRNA gene, with the forward primer 341F (CCTACGGGNGGC WGCAG) and the reverse primer 806R (GGACTACHVGGGTATCTAAT). PCR was performed using a PCR thermal cycler system (Mastercycler pro, Eppendorf, Germany). The reaction mixture (20 μL) contained 4 μL of 5 × FastPfu Buffer, 2 μL of 2.5 mM/l dNTPs, 0.4 μL of FastPfu-DNA-Polymerase, 0.8 μL of each primer and 20 ng of DNA template.

The PCR products were purified and quantified, and then sequenced at the Guangzhou Gene Denovo Company using Illumina platform (Hiseq2500 PE250). In order to quality-control, low quality sequences and chimera sequences were removed using QIIME software (version 1.9.1) and UCHIME algorithm, respectively. Then, the effective tags were clustered into Operational taxonomic units (OTUs) based on 97% similarity level by using UPARSE (version 7.0.1). Taxonomy classification of representative sequences at the phylum and genus were performed using Naïve Bayesian assignment algorithm of RDP (Version 2.2) by comparing with Silva database. Alpha diversity, including the Shannon index, Chao1 index, ACE index and Good’s coverage, and beta diversity, including the principal coordinate analysis (PCoA) was calculated using unweighted UniFrac distances. Functional prediction of microbial community was conducted using PICRUSt2. Sequence data were deposited in NCBI’s Sequence Read Archive under BioProject accession number PRJNA888371.

### Statistical analysis

The data of microbial count were log_10_-transformed. Data on the fermentation parameters of silages were analyzed by two-way ANOVA with inoculants (C, LCO, LCA, LPL, and LPE), ensiling days (7, 15, and 60 days) and their interaction as the main effects. Data on the DM and OM contents, and carbohydrate and protein fractions of 60-days fermented silages were analyzed by one-way ANOVA with inoculants as the main effect. Multiple comparisons between the means were determined by Duncan’s test. All analyses were conducted using the SAS statistical program with the level of significance set at 5%.

## Result and discussion

### Characteristics of wilted alfalfa before ensiling

The DM level, WSC content, buffering capacity and epiphytic LAB population of forage are the main factors affecting the fermentation characteristics of silage (Buxton et al., 2003). In the range of 20 to 60% DM, alfalfa silage at about 35% DM had the lowest silage pH (Kung et al., 2018). As shown in Table 1, the DM content of wilted alfalfa was 343 g/kg FW, which was a suitable level. Besides, alfalfa normally needs additional WSC for high-quality silage production compared with other forages because of its high buffering capacity (Buxton et al., 2003). In the current study, the buffering capacity and WSC content in wilted alfalfa was 322 mEq/kg DM and 49.6 g/kg DM, respectively (Table 1). The WSC content did not meet the minimum requirements for sufficient fermentation according to Pitt (1990), who reported that the minimum initial WSC content is 140 g/kg DM for wilted alfalfa at 35% DM. The starch content in wilted alfalfa was 15.4 g/kg DM, which could be used as potential fermentation substrate for lactic acid bacteria (McDonald et al., 1991; Wang et al., 2020). The nitrogen in fresh alfalfa is typically 5 to 25% NPN, but it will increase 20 to 50% after wilting (Buxton et al., 2003). In the current study, the nitrogen in wilted alfalfa contained 31.2% NPN and 68.8% TP, and more than half of the TP were intermediately degradable protein (Table 1).

**Table 1:** Chemical composition, epiphytic microbial population, and buffering capacity of wilted alfalfa before ensiling.

Item^1^	Wilted alfalfa
Dry matter (g/kg FW)	343 ± 0.73
Organic matter (g/kg DM)	890 ± 4.04
**Carbohydrates fractions (g/kg DM)**	
Neutral detergent fiber	300 ± 23.8
Acid detergent fiber	231 ± 12.1
Acid detergent lignin	48.9 ± 2.59
Water soluble carbohydrate	49.6 ± 0.70
Starch	15.4 ± 0.75
Xylose	8.63 ± 0.11
**Protein fractions**	
Crude protein (g/kg DM)	228 ± 3.35
Non-protein nitrogen (%TN)	31.2 ± 0.79
True protein nitrogen (%TN)	68.8 ± 0.78
Rapid degradation protein (%TPN)	10.1 ± 1.01
Intermediately degradable protein (%TPN)	52.0 ± 2.79
Slowly degradable protein (%TPN)	23.8 ± 1.46
Undegradable protein (%TPN)	14.0 ± 0.61
**Epiphytic microbial amount (log10 cfu/g FW)**	
Lactic acid bacteria	4.87 ± 0.16
Molds	3.94 ± 0.11
Yeasts	3.72 ± 0.13
Aerobic bacteria	5.75 ± 0.15
Buffering capacity (mEq/kg DM)	322 ± 11.3
Water activity	0.972 ± 0.002

1 CFU, colony-forming units; DM, dry matter; FW, fresh weight; TN, total nitrogen; TPN, true protein nitrogen.

The populations of epiphytic total aerobic bacteria, LAB, yeast and molds in wilted alfalfa were 5.75, 4.87, 3.72 and 3.94 log10 cfu/g FW, respectively (Table 1). The epiphytic LAB population was lower than 5 log10 cfu/g FW, which is the minimum amount to obtain well-preserved silage (Cai et al., 1999). The large number of aerobic bacteria in alfalfa, and the low population of epiphytic LAB do not ensure maximum fermentation during ensiling, this problem could be addressed by LAB inoculation.

### Dynamics of fermentation characteristics of alfalfa silage inoculated with lactobacillus

The dynamics of pH, ammonia-N, lactic acid, acetic acid and propionic acid contents and the ratio of lactic acid to acetic acid in alfalfa silages are shown in Table 2. The contents of ammonia-N, lactic acid and propionic acid, as well as pH value were significantly (*p* < 0.05) affected by ensiling days, inoculation treatments and their interaction. The ensiling days and inoculation treatments significantly (*p* < 0.05) affected acetic acid content. In the initial stage of ensiling, rapid lactic acid fermentation inhibiting harmful microbiological and plant enzymatic activity is critical for silage preservation (Shao et al., 2005; Desta et al., 2016). Numerous studies reported that the inoculation of homofermentative lactobacillus could improve the quality of silage fermentation by rapid accumulation of lactic acid (Cai et al., 1999; Slavica et al., 2015; Ni et al., 2017; Li et al., 2018). However, only LPE treatment showed lower (*p* < 0.05) pH, ammonia-N and acetic acid contents, as well as higher (*p* < 0.05) lactic acid content and the ratio of lactic acid to acetic acid than C silage during ensiling. This is related to the utilization efficiency of fermentation substrate by inoculated lactobacillus species. *L. pentosus* can ferment xylose to produce lactic acid, but *L. coryniformis*, *L. casei* and *L. plantarum* can not sufficiently utilize xylose (Liu et al., 2012; Tohno et al., 2012; Ni et al., 2015). About 60% of xylose was used in LPE silage (Tables 1, 3). However, xylose content of the other three inoculated silages was comparable to that of C silage, and their xylose consumption was about 40%. Glucose and xylose are the main monosaccharides in alfalfa, the former commonly accounts for more than 60% of WSC (Canale et al., 1991). In this study, although the xylose content of WSC in alfalfa was only about 17%, its utilization by the LAB was important in the case of insufficient fermentable glucose. In addition, silage inoculated with *L. plantarum* showed excessive acetic acid during ensiling, because it is a facultative heterofermentative LAB and tends to heterofermentation under the insufficient hexose (Buxton et al., 2003).

**Table 2:** Dynamics of fermentation characteristics of alfalfa silage inoculated with lactobacillus.

Item^1^	Ensiling days	Treatment^2^					SEM^3^	Significances^4^		
		C	LCO	LCA	LPL	LPE		D	T	D × T
pH	7	5.47^b^	5.61^a^	5.43^b^	5.61^a^	4.89^e^	0.03	***	***	***
	15	5.45^b^	5.39^bc^	5.57^ab^	5.53^ab^	4.87^e^				
	60	5.35^bc^	5.19^d^	5.30^c^	5.32^bc^	4.87^e^				
Ammonia-N (g/kg TN)	7	77.7^c^	96.8^b^	78.6^c^	84.5^bc^	35.1^e^	3.85	***	***	**
	15	112^ab^	104^ab^	109^ab^	111^ab^	62.2^d^				
	60	120^a^	109^ab^	119^a^	121^a^	66.8^cd^				
Lactic acid (g/kg DM)	7	16.5^e^	14.5^ef^	16.0^e^	12.7^f^	26.2^b^	1.01	***	***	***
	15	20.3^d^	22.7^c^	18.5^de^	21.0^cd^	31.7^a^				
	60	18.4^de^	26.7^b^	21.0^cd^	21.7^cd^	31.4^a^				
Acetic acid (g/kg DM)	7	19.9^f^	25.1^d^	22.5^e^	28.8^c^	16.3^g^	0.88	***	***	ns
	15	27.1^cd^	30.5^b^	30.6^b^	32.7^ab^	19.9^f^				
	60	29.6^bc^	31.5^b^	31.3^b^	34.3^a^	23.2^de^				
Propionic acid (g/kg DM)	7	0.38^d^	0.37^d^	0.38^d^	0.40^d^	0.42^d^				
	15	1.53^a^	1.27^b^	1.23^bc^	0.98^c^	1.00^c^	0.04	***	*	***
	60	1.58^a^	1.25^bc^	1.27^b^	1.30^b^	1.49^ab^				
Lactic acid / acetic acid	7	0.83^c^	0.58^d^	0.71^cd^	0.44^e^	1.60^a^	0.12	ns	***	**
	15	0.75^cd^	0.74^cd^	0.61^d^	0.64^d^	1.59^a^				
	60	0.63^d^	0.85^c^	0.67^cd^	0.63^d^	1.34^b^				

1 DM, dry matter; TN, total nitrogen. Butyric acid was not detected.

2 C, control; LCO, *Lactobacillus coryniformis*; LCA, *Lactobacillus casei*; LPL, *Lactobacillus plantarum*; LPE, *Lactobacillus pentosus*.

3 SEM, standard error of means.

4 D, ensiling days; T, inoculum treatments; D × T, the interaction between ensiling days and inoculum treatments.

*p < 0.05; **p < 0.01; ***p < 0.001. Means with different letters in the same analysis indicated a significant difference ns, not significant.

**Table 3:** Effect of lactobacillus on chemical composition and lactic acid bacteria populations of alfalfa silages after 60 days ensiling.

Item^1^	Treatment^2^					SEM^3^	Significances
	C	LCO	LCA	LPL	LPE		
Dry matter (g/kg FW)	351^b^	353^ab^	349^b^	346^b^	363^a^	1.04	*
Organic matter (g/kg DM)	893	894	894	892	897	0.60	ns
**Carbohydrates fractions**							
Neutral detergent fiber (g/kg DM)	356	355	359	349	339	1.69	ns
Acid detergent fiber (g/kg DM)	292	283	282	277	270	1.81	ns
Acid detergent lignin (g/kg DM)	66.6^a^	69.6^a^	70.5^a^	69.7^a^	49.8^b^	2.94	*
Water soluble carbohydrate (g/kg DM)	12.4	11.8	12.3	11.0	10.2	0.32	ns
Starch (g/kg DM)	7.49	6.93	7.36	7.59	7.63	0.24	ns
Xylose (g/kg DM)	5.12^ab^	4.81^b^	5.55^a^	5.31^a^	3.44^c^	0.14	***
**Protein fractions**							
Crude protein (g/kg DM)	219	223	218	224	224	1.03	ns
Non-protein nitrogen (%TN)	74.6	74.3	74.7	76.3	76.7	0.74	ns
Ammonia nitrogen (%NPN)	16.5^a^	15.1^a^	16.0^a^	15.8^a^	8.72^b^	0.66	***
True protein nitrogen (%TN)	25.4	25.7	25.3	23.7	23.3	0.74	ns
Buffer soluble protein (%TPN)	6.65^b^	7.43^b^	9.26^ab^	10.4^a^	10.8^a^	0.49	*
Neutral detergent soluble protein (%TPN)	56.2	56.8	55.5	53.2	53.6	1.07	ns
Acid detergent soluble protein (%TPN)	8.56^b^	8.99^ab^	7.49^b^	10.3^ab^	11.1^a^	0.31	*
Acid detergent insoluble protein (%TPN)	28.5^a^	26.8^ab^	27.7^ab^	26.1^ab^	24.5^b^	0.77	*
Lactic acid bacteria (log10 cfu/g FW)	7.42	7.40	7.46	7.57	7.63	0.16	ns

1 DM, dry matter; FW, fresh weight; NPN, non-protein nitrogen; TN, total nitrogen; TPN, true protein nitrogen.

2 C, control; LCO, *Lactobacillus coryniformis*; LCA, *Lactobacillus casei*; LPL, *Lactobacillus plantarum*; LPE, *Lactobacillus pentosus*.

3 SEM, standard error of means.

*p < 0.05; **p < 0.01; ***p < 0.001. Means with different letters in the same row indicated a significant difference ns, not significant.

The contents of ammonia-N and butyric acid are the critical index to determine the fermentation quality of silage. These two variables should be less than threshold of 10% TN and 0.2% DM, respectively, in well-preserved silage (Catchpoole and Henzell, 1971). According to this criterion, all silages except LPE treatment did not obtain good fermentation quality. Although butyric acid was not detected, their ammonia-N content exceeded the threshold after 15 days ensiling. Butyric acid was absent in all silages after wilting, which was consistent with the report of Zhang et al. (2020). Accumulation of butyric acid is associated with clostridium activity during ensiling. Under neutral conditions, the optimum and the minimum water activity for clostridium growth were 0.995 and 0.971, respectively, and the minimum water activity increased with the pH declined. Moreover, clostridia are more susceptible to water activity than LAB, the latter are the most tolerant of low water activity, about 0.930 to 0.945 (Buxton et al., 2003). In this study, the water activity of wilted alfalfa was about 0.972. Clostridium growth may be completely suppressed after wilting, supported by the absence of butyric acid detection in all silages.

### Effects of lactobacillus inoculation on carbohydrate and nitrogenous fractions of alfalfa silage

Lactobacilli inoculants had no significant effect on carbohydrate fractions, such as WSC, starch, NDF and ADF contents in alfalfa silage (Table 3). Oliveira et al. (2017) summarized 1747 literatures and pointed out that silage inoculated with homofermentative and facultative heterofermentative LAB could reduce WSC content and promote lactic acid production in silage, but did not affect NDF content. In this study, the residual WSC content did not show a significant difference (*p* > 0.05) among all silages. This might be due to insufficient WSC content of fresh forages, which were fully utilized by LAB during ensiling. However, LPE silage had the lowest xylose content (*p* < 0.001) among all silages, which was due to the fact that *L. pentosus* can ferment xylose to produce lactic acid. Most strains of LAB do not hydrolyze starch and cellulose (McDonald et al., 1991). Therefore, there were no differences (*p* > 0.05) in the starch, NDF and ADF contents among all silages. In addition, LPE silage had lower (*p* < 0.05) ADL content and higher (*p* < 0.05) DM content than that of C silage. The ADL is difficult to degrade during ensiling, and its content is negatively correlated with dry matter loss (Guo et al., 2014). Thus, this might be related to the good quality of LPE silage, since high quality silage usually has low dry matter loss (Buxton et al., 2003).

In contrast to carbohydrate fractions, ensiling did not affect the crude protein content of forage, but large amounts of TP are decomposed into NPN during ensiling. Proteolysis in silage can be inhibited by LAB inoculation (Tao et al., 2017; Guo et al., 2020). In the current study, silage inoculated with lactobacillus had no significant effect on the CP content and the proportion of NPN, but the proportion of ammonia-N in LPE silage decreased (*p* < 0.05) compared to C silage (Table 3). This is due to the rapid decrease in silage pH, which inhibits further decarboxylation or deamination of amino acids by harmful microorganisms (Buxton et al., 2003). Compared with C silage, lactobacilli inoculants tended to increase the proportion of buffer soluble protein and acid detergent soluble protein in TP. The research by Tao et al. (2017) showed similar changes in silage protein fractions after LAB inoculation.

### Effects of lactobacillus inoculation on the bacterial community in alfalfa silage

The alpha-diversity index of alfalfa silage inoculated with or without lactobacillus are shown in Table 4. The Goods_coverage index of each group was over 0.99, indicating that the sequencing analysis could reflect the real bacterial community composition in silage samples. Alfalfa silage inoculated with *L. pentosus* had lower (*p* < 0.05) Shannon, Chao1 and ACE index than that of C treatment, indicating that the bacterial diversity was reduced. This was due to the fact that the decreased pH resulted from LAB inoculation inhibited the growth of acid-resistant bacteria and thus reduced the bacterial diversity in silage (Zhao et al., 2021).

**Table 4:** Alpha-diversity of bacterial community in alfalfa silage inoculated with lactobacillus.

Item	Treatment^1^					SEM^2^	Significances
	C	LCO	LCA	LPL	LPE		
Shannon	5.47^a^	5.55^a^	4.96^ab^	4.99^ab^	4.80^b^	0.09	***
Chao 1	819^a^	759^b^	809^a^	799^ab^	746^b^	8.6	*
ACE	829^a^	752^b^	808^a^	787^ab^	750^b^	8.7	*
Goods_coverage	0.997	0.997	0.997	0.997	0.998	0.001	ns

1 C, control; LCO, *Lactobacillus coryniformis*; LCA, *Lactobacillus casei*; LPL, *Lactobacillus plantarum*; LPE, *Lactobacillus pentosus*.

2 SEM, standard error of means.

*p < 0.05; **p < 0.01; ***p < 0.001. Means with different letters in the same row indicated a significant difference ns, not significant.

**Figure 1:**
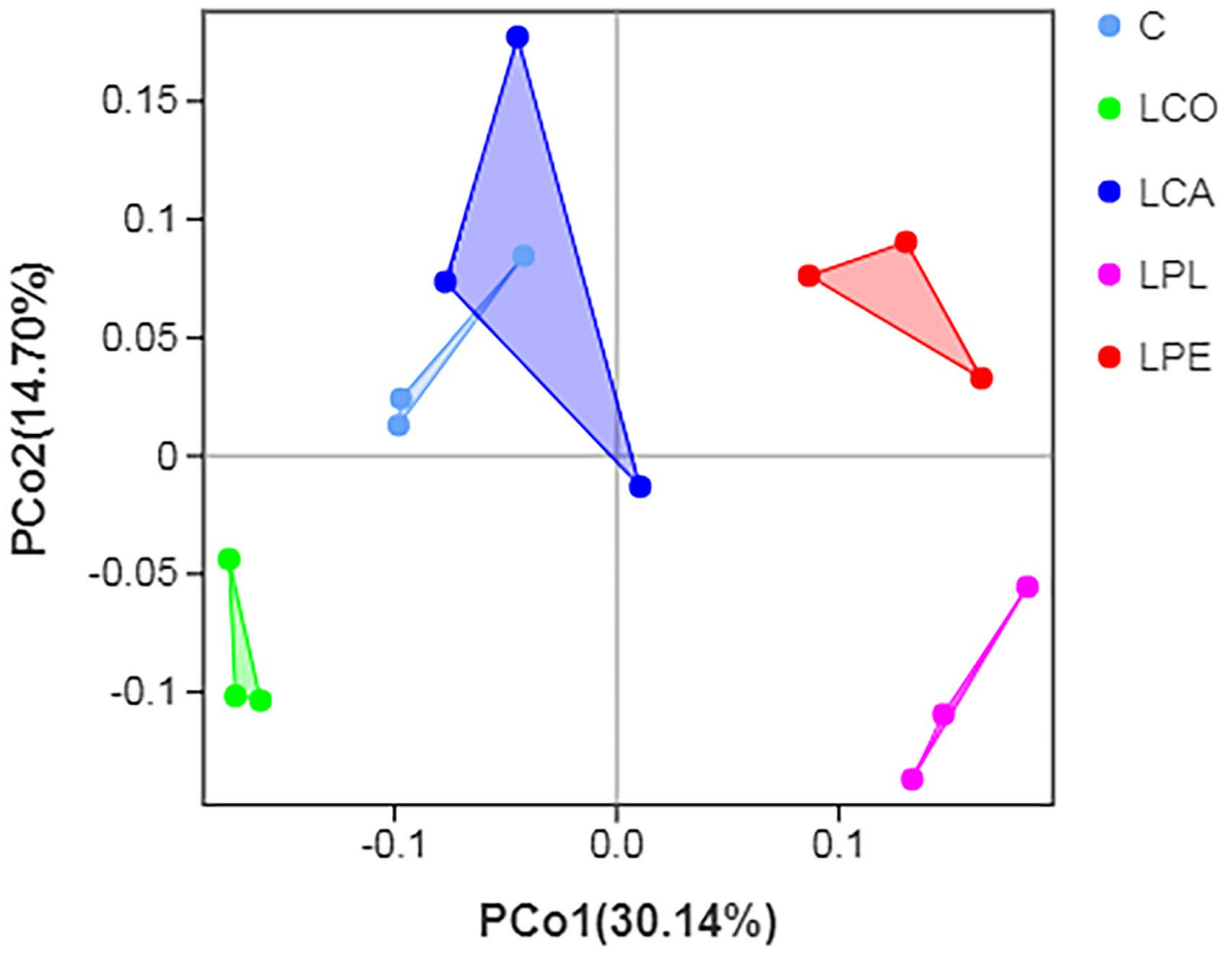
Differences in microbial community diversity on genus level shown by PCoA. C, control; LCO, *Lactobacillus coryneformis*; LCA, *Lactobacillus casei*; LPL, *Lactobacillus plantarum*; LPE, *Lactobacillus pentosus*.

The PCoA of bacterial community in alfalfa silage inoculated with or without lactobacillus is shown in Figure 1. Samples from each treatment were individually clustered within a narrow range, indicating good sample repeatability. The distribution and composition of microbial communities were significantly separated and varied across treatments. The C and LCA group samples were mainly clustered in the upper left quadrants. However, the other three groups were completely separated from C and LCA group, with the LPE, LPL and LCO group samples locating in the upper right, lower right, and lower left quadrants, respectively. This result indicated that the *L. coryneformis*, *L. plantarum* and *L. pentosus* inoculants could significantly affected microbial composition compared *L. casei*. Similarly, Yang et al. (2019) found that of the *L. plantarum* inoculants could markedly affect the bacterial community composition of alfalfa silage.

**Figure 2:**
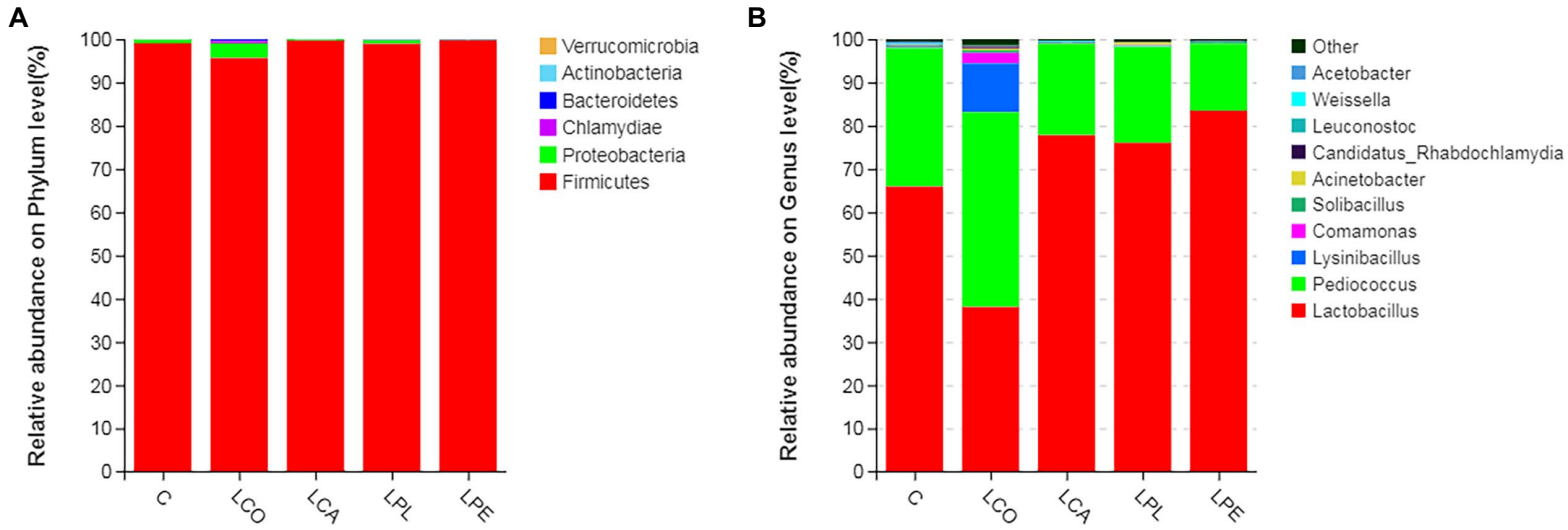
Relative abundance of bacterial community by phylum **(A)** and genus **(B)** for alfalfa silage. C, control; LCO, *Lactobacillus coryneformis*; LCA, *Lactobacillus casei*; LPL, *Lactobacillus plantarum*; LPE, *Lactobacillus pentosus*.

The relative abundance of bacterial community in alfalfa silage at the phylum level is shown in Figure 2A. In general, the dominant phylum was *Firmicutes*, followed by *Proteobacteria* in all silages. McGarvey et al. (2013) reported that the relative abundance of *Firmicutes* in alfalfa silage usually more than 95%. In the current study, the relative abundance of *Firmicutes* in alfalfa silage after 60 days ensiling was more than 99%, except LCO group (about 95%). *Proteobacteria* are usually the dominant phylum in fresh forage before ensiling, and the bacterial community shifts from *Proteobacteria* to *Firmicutes* during ensiling (Keshri et al., 2018). Ni et al. (2018) reported that the major genera related to silage fermentation were *Lactobacillus*, *Pediococcus*, *Weissella*, and *Leuconostoc*, all of which belonged to *Firmicutes*. As exhibited in Figure 2B, the prevalent genera in alfalfa silage were *Lactobacillus* and *Pediococcus* in this study. The relative abundance of *Lactobacillus* in alfalfa silage were 83.5, 77.8, 76.0, 65.9 and 38.1% in LPE, LCA, LPL, C and LCO group, respectively. The relative abundance of *Pediococcus* in alfalfa silage was in the following order: LCO (45.0%) > C (32.0%) > LPL (22.2%) > LCA (21.1%) > LPE (15.4%). Generally, *Pediococcus* usually grow vigorously and initiate lactic acid fermentation in the early stage of silage, while they will be gradually replaced by *Lactobacillus* as the pH decreases (Ni et al., 2017). In present study, the highest relative abundance of *Lactobacillus* and lowest relative abundance of *Pediococcus* in LPE silage corresponded to the lowest silage pH. However, higher relative abundance of *Pediococcus* in the LCO group may be relative to the postponed formation of acidic environment (Yang et al., 2019). The relative abundance of *Weissella* and *Leuconostoc* were extremely minor in all silages, which were in consistent with the finding of Ogunade et al. (2018), who concluded that silage inoculated with LAB could decrease the relative abundance of *Weissella*. It is interesting to note that the *Lysinibacillus* and *Comamonas* were only found in the LCO silage. However, researchers reported that the *Lysinibacillus* and *Comamonas* could not assimilate and ferment sugars (Ni et al., 2013).

**Figure 3:**
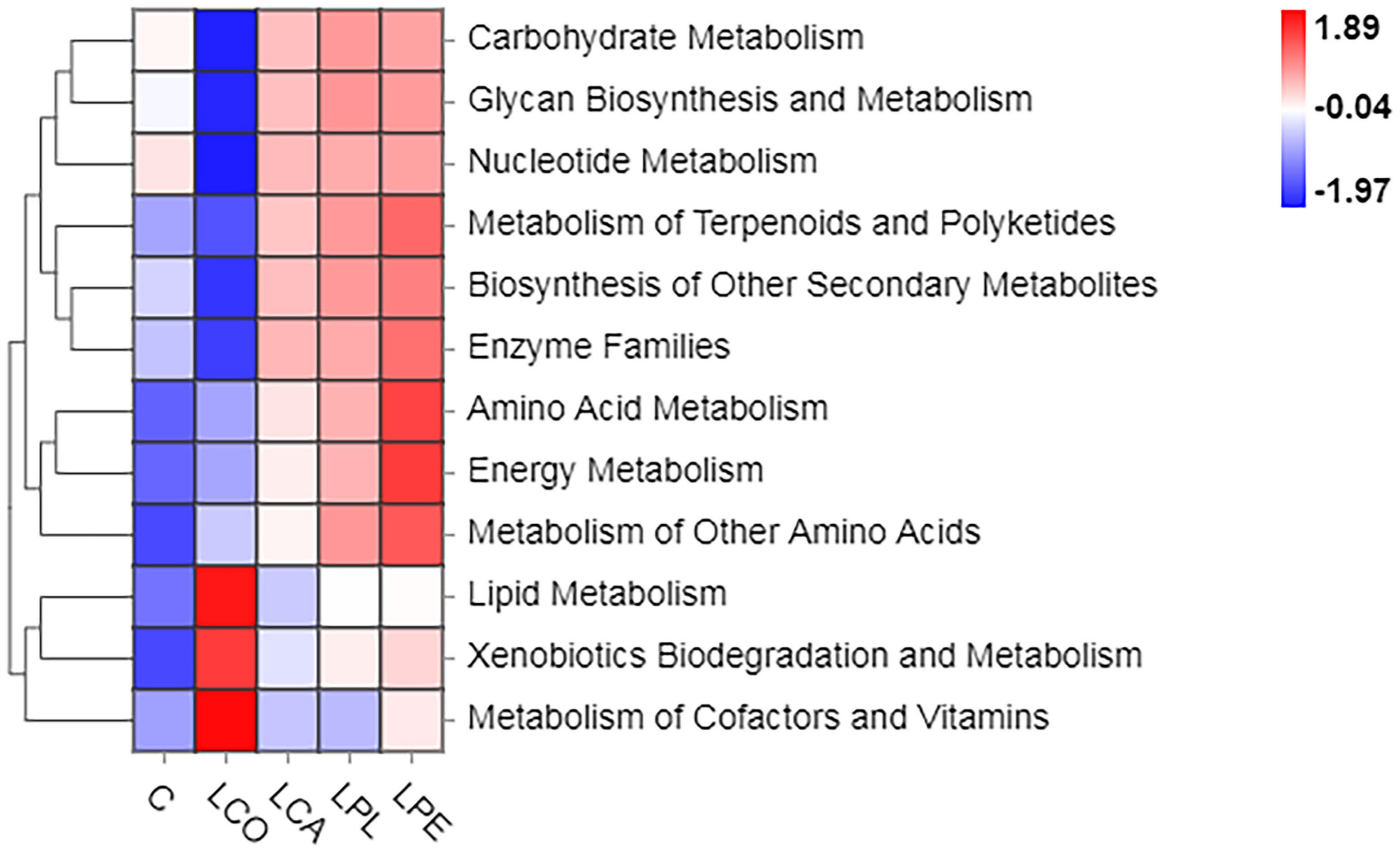
Metabolic functional analysis of microbial community using PICRUSt2. C, control; LCO, *Lactobacillus coryneformis*; LCA, *Lactobacillus casei*; LPL, *Lactobacillus plantarum*; LPE, *Lactobacillus pentosus*.

The functional prediction of microbial community in alfalfa silage inoculated with or without lactobacilli based on 16S rDNA gene are shown in Figure 3. Compared with the C silage, the metabolism of carbohydrate, glycan, nucleotide, terpenoids and polyketides, amino acid and energy, and biosynthesis of other secondary metabolites were up-regulated by LCA, LPL, or LPE treatments, especially LPE treatment, which significantly up-regulated amino acid, carbohydrate, energy, terpenoids and polyketides metabolism. Previous studies have reported that the decrease in ammonia-N content is accompanied by the down-regulation of amino acid metabolism of microbial community in good silage (Bai et al., 2022). The results of the LPE treatment did not fit this theory, with the highest level of amino acid metabolism and the lowest ammonia-N content among all treatments. However, the LPE treatment also had high buffer soluble protein contents and low acid detergent insoluble protein. This might be due to the fact that the large degradation of amino acids and small peptides caused by harmful microorganisms, such as clostridium and enterobacter, did not occur during proteolysis (Yuan et al., 2020). In addition, the up-regulated energy, terpenoids and polyketides metabolism in LPE silage, which was consistent with the report of Xu et al. (2019), who found that the energy metabolism was up-regulated when silage inoculated with LAB. In present study, it was interesting to note that carbohydrate, glycan and nucleotide metabolism was down-regulated and lipid, xenobiotics biodegradation, cofactors and vitamins metabolism was up-regulated in the LCO treatment compared with the C treatment. Limited studies have found that *L. coryniformis* has an inhibitory effect on clostridium and yeast by producing bacteriocins (Tanaka et al., 2009; Slavica et al., 2015). These bacteriocins might inhibit microbial growth by reducing its carbohydrate, glycan and nucleotide metabolism. Meanwhile, the *L. coryniformis* inoculants might enhance its biodegradation activities by mobilizing the metabolism of lipid and cofactors and vitamins to remove the xenobiotics.

## Conclusion

The *L. pentosus* inoculants enhanced lactic fermentation, which had the highest lactic acid content and lowest silage pH, acetic acid and ammonia-N contents, and thus improved fermentation quality of alfalfa silage. The residual WSC content was not different among all silages, *L. pentosus* inoculants was more efficient at using xylose to produce lactic acid, with lower xylose and acetic acid contents and higher lactic acid content in the silage than the other LAB inoculants. The *L. coryniformis*, *L. casei* and *L. plantarum* inoculants had limited effect on fermentation quality of alfalfa silage. Silage inoculated with lactobacillus tended to increase the proportion of buffer soluble protein and acid detergent soluble protein in TP. Compared with the control, *L. pentosus* inoculants reduced the bacterial diversity in alfalfa silage, with lower Shannon, Chao 1 and ACE indices, and promoted relative abundance of lactobacillus and decreased the relative abundance of *Pediococcus*. As well as *L. pentosus* inoculants up-regulated amino acid, carbohydrate, energy, terpenoids and polyketides metabolism, and promoted lactic acid fermentation process. In summary, *L. pentosus* used as an additive was superior to the other three lactobacillus species in improving fermentation quality of alfalfa silage.

## Data availability statement

The datasets presented in this study can be found in online repositories. The names of the repository/repositories and accession number (s) can be found at: https://www.ncbi.nlm.nih.gov/, PRJNA888371.

## Author contributions

WH wrote the manuscript. GG designed the study. WH, YZ, LZ, CS, and LC performed the experiments. QL, SZ, CW, and GG conducted the statistical data and bioinformatics analyses. All authors contributed to the article and approved the submitted version.

## Funding

This work was supported by the grant for National Natural Science Foundation of China (nos. 32001405 and 31502015) and Animal Husbandry Key Discipline construction program in “1331 project” of Shanxi Province.

## Conflict of interest

The authors declare that the research was conducted in the absence of any commercial or financial relationships that could be construed as a potential conflict of interest.

## Publisher’s note

All claims expressed in this article are solely those of the authors and do not necessarily represent those of their affiliated organizations, or those of the publisher, the editors and the reviewers. Any product that may be evaluated in this article, or claim that may be made by its manufacturer, is not guaranteed or endorsed by the publisher.
